# Corrosion Product Film of a Medium-Mn Steel Exposed to Simulated Marine Splash Zone Environment

**DOI:** 10.3390/ma14195652

**Published:** 2021-09-28

**Authors:** Xinyong Yan, Shumei Kang, Meiling Xu, Pengyu Li

**Affiliations:** School of Materials and Metallurgy, University of Science and Technology Liaoning, Anshan 114051, China; yan1179316467@163.com (X.Y.); xumeiling1748@163.com (M.X.); lipengyu927@163.com (P.L.)

**Keywords:** medium-Mn steel, marine splash zone, electrochemistry, corrosion behavior, corrosion product film

## Abstract

The corrosion behavior of a medium-Mn steel in a simulated marine splash zone was studied by a dry–wet cyclic corrosion experiment and electrochemical experiment. The corrosion products were characterized by corrosion rate calculation, composition detection, morphology observation, element distribution detection, valence analysis, polarization curve, and electrochemical impedance test. The results show that the corrosion products of the sample mainly include γ-FeOOH, Fe_x_O_y_, Mn_x_O_y_, and a small amount of (Fe,Mn)_x_O_y_, and the valence state of iron compounds and manganese compounds in different corrosion stages changed obviously. In the initial corrosion products, Mn is enriched significantly and facilitates the electrochemical reaction of corrosion process. The content of Ni in the inner rust layer is high. The semi-quantitative analysis of the corrosion product elements shows that the atomic concentrations of Cr and Mo increase significantly in later corrosion products, indicating that the dense isolation layer formed by alloy element compounds in the corroded layer is the main factor to improve the protection ability of the rust layer at the end corrosion stage of the sample. With the corrosion durations, the corrosion current density of the sample with the corrosion product film first increases and then decreases, and the corrosion potential first moves negative and then shifts in a positive direction subsequently, indicating that the protective effect of the corrosion product film is gradually significant.

## 1. Introduction

With the global over-exploitation of terrestrial resources and the continuous depletion of energy sources, the move to marine resources has become a new development trend [1,2,3]. Considering the economy and reliability of offshore platform equipment, a medium-Mn steel with composition specifications of 0.267C-4.390Mn-0.020Cu-0.385Cr-0.345Mo-3.270Ni mass% is newly developed for offshore industrial platforms [4,5]. On the one hand, increasing Mn and other alloy elements to a certain extent will damage the casting properties of steels [6,7], but replacing Ni, Mo, and other elements with relatively cheap alloying element Mn can greatly reduce the casting cost of steel [8,9]. On the other hand, its superior mechanical properties, high strength, and good low-temperature toughness can meet the comprehensive mechanical properties of marine equipment structures [10,11]. It is well known that the marine splash zone has a harsh corrosive environment, and the offshore platform steel is the most widely used in this region. High concentrations of dissolved oxygen, sufficient light, and wind waves cause serious corrosion on the surface of steel structures exposed to seawater here [12,13,14]. In the past, some experimental studies have attempted to elucidate the corrosion mechanism of steel in the splash zone [15,16,17,18]. For example, some studies have emphasized that high concentrations of chloride ions lead to rust cracking [19]. Chen et al. [20] reported that the cation selective permeability of the rust layer led to the anion concentration gradient of the whole rust layer in industrial and non-industrial marine splash zones environment. Sulfur dioxide could promote the formation of a-FeOOH, which resulted in the formation of obvious anode and cathode regions at the rust/steel interface. Zhang et al. [21,22] conducted a simulated seawater corrosion study on low-carbon medium-Mn steel and found that the corrosion products in the marine splash zone environment consisted mainly of manganese compounds, FeOOH and FexOy, in which manganese compounds increased the defect density of corrosion products and deteriorated the protection ability of corroded layer. However, the influence and mechanism of rust layers in different corrosion stages as well as alloy elements Mn, Ni, Cr, and Mo on the corrosion behavior of medium-Mn in a splash zone corrosion environment have not been systematically elucidated. Therefore, in this study, the corrosion behavior of tested steel in a simulated marine splash zone was studied by dry–wet cyclic corrosion experiment. The polarization curves and electrochemical impedance spectra of the rust layers formed in each corrosion stage were further investigated by electrochemical workstation, and the influence of corrosion product film on the corrosion process was explored.

## 2. Materials and Methods

### 2.1. Material Preparation

The chemical compositions of tested medium-Mn steel in mass% were C 0.267, Mn 4.390, Cr 0.385, Cu 0.020, Ni 3.270, Mo 0.345, Si 0.260, and Fe balance. All samples were machined by the wire cutting machine tool (DK7735, Shibeite CNC machine tool factory, Taizhou, China) into two sizes: 60 mm × 40 mm × 4 mm and 10 mm × 10 mm × 4 mm; to facilitate the hanging of specimen, a hole with a radius of 1.5 mm was drilled on each sample. The large sample was used to calculate the corrosion rate by weight loss test (three parallel samples per test period), and the small sample was used for corrosion products detection and an electrochemical test (four parallel samples per test period). Samples used for electrochemical testing are soldered with copper wires on their non-testing surfaces and then encapsulated with epoxy resin on the rest of the surface, leaving only a testing surface of 1 cm^2^. Before the experiment, all the samples were polished to 2000# SiC sandpaper, and then the samples were cleaned with acetone, absolute ethanol, and deionized water under ultrasonic vibration.

### 2.2. Wet–Dry Cyclic Corrosion Experiment

Dry–wet cyclic corrosion experiments were used to simulate the corrosion effects of the test steel in the corrosive environment of the marine splash zone. (The experimental equipment for simulating the corrosion environment of the marine splash zone is patented as ZL 2020 2 1337994.3) The samples were subjected to cyclic corrosion for 24, 72, 168, 288, 432, and 600 h in dry/immersion environments. Each dry–wet cycle included a 10-minute immersion stage and a 50-minute drying stage. The immersion period: 3.5% NaCl solution, temperature is 25 °C, PH is 6.5. The drying period: temperature is 27 °C, RH is 47%. For samples for corrosion kinetics calculations, weighing before the experiment, and then at room temperature, the rust layer was removed by a chemical cleaning solution mixed with 37 (vol) % hydrochloric acid (HCl, GR), deionized water, and hexamethylenetetramine (C_6_H_12_N_4_, AR, ≥99.0%) at 100:100:1. The derusted samples were washed by absolute ethanol (C_2_H_6_O, AR, ≥99.7%), dried, and then weighed again after one day. 

### 2.3. Electrochemical Testing

The samples with corrosion product film after dry–wet cyclic corrosion for different times were tested by an electrochemical workstation (AUTOLAB PGSTAT302N2). A three-electrode system was adopted with platinum as the auxiliary electrode (CE), KCl-saturated calomel electrode as the reference electrode (RE), samples with a rust layer of 1 cm^2^ area after dry/wet alternate corrosion for 24, 72, 168, 288, 432, and 600 h were used as working electrodes, and the electrolyte is 3.5% sodium chloride corrosion solution. The sample steel was put into electrolyte solution for 1 h open circuit potential detection; when the open circuit potential reaches a stable value, the samples were tested for their electrochemical impedance spectra in the frequency range of 10^5^ to 10^−2^ Hz at this potential (to be subjected to a system 10 mV sine wave AC interference signal). When measuring the polarization curve, the scanning range is from −1.5 to 0.5 V (versus ***E***_oc_) and the scanning rate of the system is 0.5 mV/s. All the measurements of samples were carried out at room temperature (25 ± 1 °C). 

### 2.4. Characterization of Corrosion Products

The microstructure morphology of the sample was observed by optical microscope (OM, ZEISS AXIO VERT, A1, Carl Zeiss, Jena, Germany). The composition of corrosion products was detected by X-ray diffractometer (XRD, D8 ADVANCE, Bruker, Germany) equipped with CuKa radiation (λ = 0.154 nm), the scanning step is 0.028, and the scanning angle is 10–70°. The surface morphology of the rust layer was observed by field emission high-resolution scanning electron microscope (SEM, SIGMA HD, Carl Zeiss, Jena, Germany). The test steels with a corrosion product layer were embedded into the conductive resin to display the cross-section morphology. The cross-section morphology of corrosion was observed by scanning electron microscope, and the distribution of elements was detected by energy dispersive X-ray spectrometer (EDS, JSM-6390A, Joint-stock Company, Beijing, China). The valence states of elements were analyzed by X-ray photoelectron spectroscopy (XPS, K-AIPHA, Thermo Scientific, Oxford, UK).

## 3. Results and Discussion

### 3.1. Metallographic Organization

**Figure 1:**
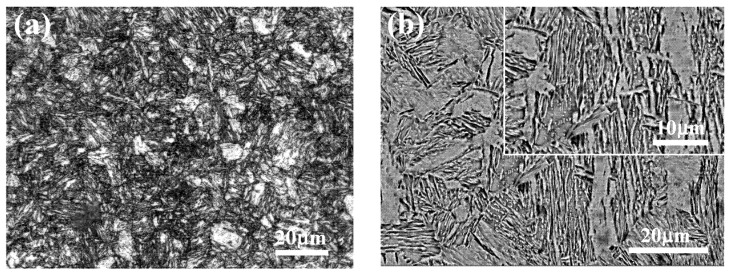
Metallographic structure of sample: (**a**) OM micrograph; (**b**) SEM micrograph.

The metallographic structure of test steel is shown in Figure 1. The organization structure of medium-Mn steel consists of lath-like tempered martensite and reversed austenite. The reversed austenite distributes along the boundary of tempered martensite and refines the lath-like martensite, which makes the microstructure of medium-Mn steel fine and uniform, and it has good strength as well as toughness.

### 3.2. Corrosion Kinetics

The corrosion kinetics of sample steels were determined by the weight loss method, the mass loss of the samples measured before and after corrosion is brought into the following formula (1) for calculation:

(1) $$\begin{array}{l} CR\;\left[\frac{mm}{\mathrm{Year}}\right]=\frac{mass\;loss\;\left[g\right]\times 8760\;\left[h/\mathrm{year}\right]}{corrosion\;time\;\left[h\right]\times metal\;density\;\left[\frac{g}{{\mathrm{cm}}^{3}}\right]\times exposed\;area\;\left[{\mathrm{cm}}^{2}\right]}\times \frac{10\;\left[\mathrm{mm}\right]}{1\;\left[\mathrm{cm}\right]}\\ \mathrm{simplifying}:\;CR\;\left[\frac{\mathrm{mm}}{\mathrm{Year}}\right]=\frac{\Delta m\times 87,600}{t\rho s} \end{array}$$

where *CR* is the average annual corrosion rate (mm/y), Δ*m* is the mass loss (g), *t* is the corrosion time (h), *ρ* is the density of tested steel (7.90 g/cm^3^), and ***S*** is the exposed area of the test specimen (cm^2^).

**Figure 2:**
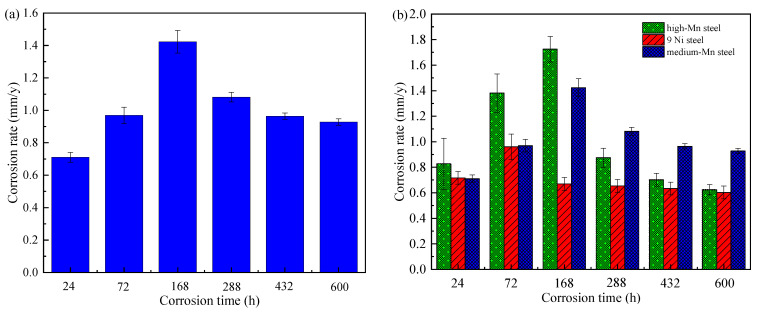
Average annual corrosion rates variation of test steels: (**a**) medium-Mn steel; (**b**) medium-Mn and comparison steels.

The relationship between the average annual corrosion rate and corrosion time is shown in Figure 2. The average annual corrosion rate shows a trend of first increase and then decrease. In the initial corrosion stage, the corrosion rate is showing a rapid upward trend, due to the initial stage of the rust layer being very thin, the corrosive ions in the medium attaching directly to the surface of the substrate diffusion, and the substrate occurring in a strong electrochemical reaction. The cathodic process includes oxygen reduction and rust layer component reduction, the strong or weak change of these two effects is the main reason for the increase in the initial average annual corrosion rate. After 168 h of corrosion, the corrosion rate of the medium-Mn steel reached a peak; since the corrosion lasted for a period of time, the rust layer that formed on the substrate surface blocked the direct contact between the steel matrix and the corrosive solution, and the turning of velocity is caused by the compactness or physicochemical properties of rust layer. In the corrosion 288 h later, the corrosion rate tends to level off and stabilize. It means that compared with the corrosion layer in the previous stages, the corrosion layer formed by the accumulation of corrosion products in this stage began to protect the steel matrix. In order to better reflect and evaluate the protective performance of the corrosion product film, we carried out a comparative experiment with two other test steels (high-Mn steel: ZGMn13-4, 9Ni steel: 9Ni590B) under the same conditions. The average annual corrosion rates of the three test steels are shown in Table 1. The results show that the corrosion rate of medium-Mn steel in the test is low in the early corrosion stage, and the corrosion rate in the middle/late stage of corrosion is significantly lower than that in the early stage of corrosion. However, compared with the other two contrast samples, the corrosion rate of the test medium-Mn steel is higher, and the protection of the corrosion product film is relatively poor.

### 3.3. Corrosion Products Composition

**Figure 3:**
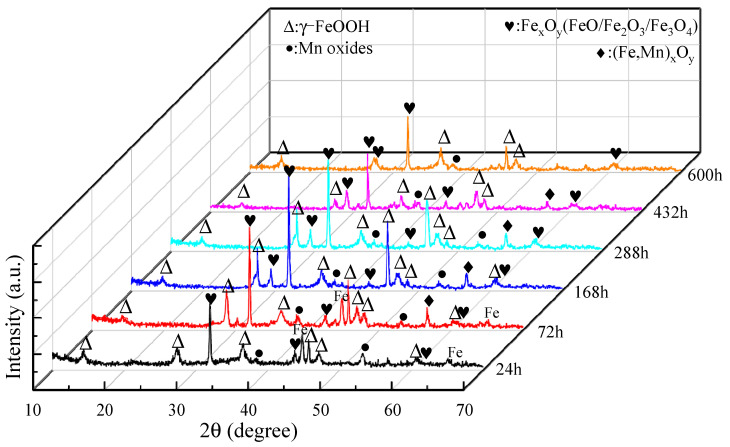
XRD diffraction patterns of corrosion products.

The XRD diffraction patterns of the corrosion products of the samples after corrosion at different times are shown in Figure 3. The results show that the corrosion products of medium-Mn steel are in addition to the basic components of lepidocrocite (γ-FeOOH), iron oxides (FeO/Fe_2_O_3_/Fe_3_O_4_), and manganese oxides (MnO_2_/Mn_3_O_4_); a small amount of iron-manganese oxides (Fe,Mn)_x_O_y_) also appear in the middle/late stages of corrosion. When the corrosion time is prolonged, the composition of corrosion products evolved. The corrosion products after 24 h and 72 h corrosion appear as the strong diffraction peaks of Fe, which is because the rust layer is thin at the initial stage of corrosion and is easy to be penetrated by X-rays. At the early stage of corrosion, the corrosion products were mainly FeO, Fe_2_O_3_, MnO_2_, and a large amount of γ-FeOOH; ferrous oxide is poorly stable, and manganese oxide has a special porous structure and adsorption properties. γ-FeOOH is a semiconductor. In addition to its special anion selectivity, it also has strong electrochemical activity [23,24,25], which makes the stability of the rust layer very poor, and corrosive ions can easily pass through the rust layer and act on the matrix. In the middle period of corrosion, in the rust layer, there appears a small amount of iron-manganese oxide (Fe,Mn)_x_O_y_), which will not only reduce the denseness of the outer rust layer so that ions to the inner layer diffuse but also adsorb anions in the medium. Therefore, the samples at this stage also showed a high corrosion rate. In the later corrosion products, the number of diffraction peaks of manganese oxides and γ-FeOOH decreases, and iron oxides mainly exist in the form of Fe_2_O_3_ and Fe_3_O_4_. Fe_3_O_4_ is an electrical conductor with good denseness and strong stability [26,27], which can improve the protective effect of the corrosion product film. 

### 3.4. Surface and Cross-Section Morphology of Corrosion Products

**Figure 4:**
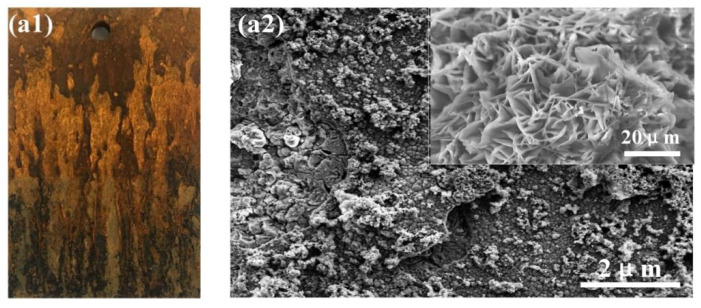

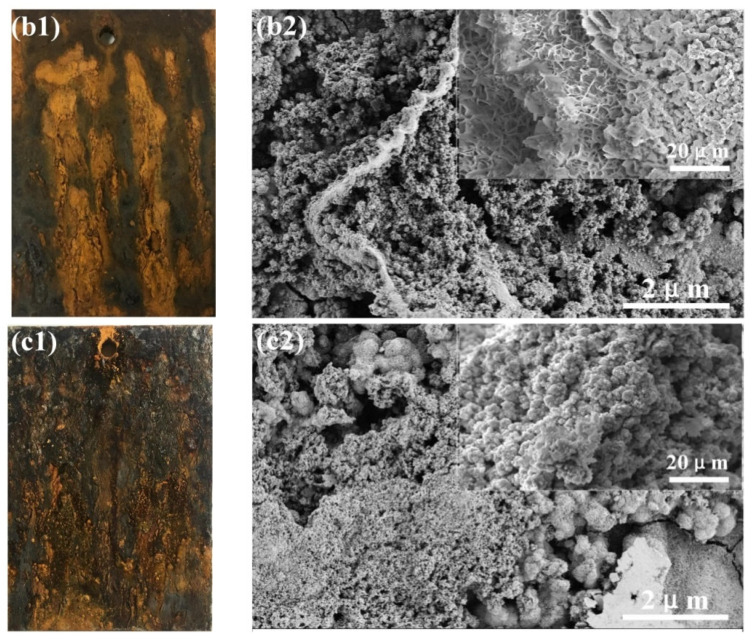
Surface macro (**a1**–**c1**) and micro (**a2**–**c2**) morphology of corrosion products of medium-Mn steel at different corrosion stages: (**a1**,**a2**) 72 h; (**b1**,**b2**) 288 h; (**c1**,**c2**) 600 h.

Figure 4 shows the surface micro and macro morphology of the corrosion products of medium-Mn steel. As shown in the figure, with the extension of corrosion time, the color of the rust layer of the sample gradually changed from light yellow to yellowish brown. The color of the rust layer near the substrate side was darker, and the black corrosion was thicker and denser in the later stage of corrosion, while the uplifted light yellow rust layer gradually decreased. After 72 h of corrosion, the surface of the rust layer presents snowflake-shaped and lamellar morphology, and the flakes are very loose. When the corrosion testing time is prolonged, the substrate surface corrosion products accumulate and evolve. After 288 h of corrosion, the surface morphology began to evolve from lamellae to large flattened clusters, and the flattened clusters were significantly denser than the rust layer morphology of previous stage. After 600 h of corrosion, the cluster-like rust layer continues to grow and evolve into thick pellets, with stacked distribution between pellets, and large holes and gaps are rarely observed. The surface morphology of corrosion products appears dense and thick, indicating that the corrosion product film has a protective ability to steel matrix at this stage.

**Figure 5:**
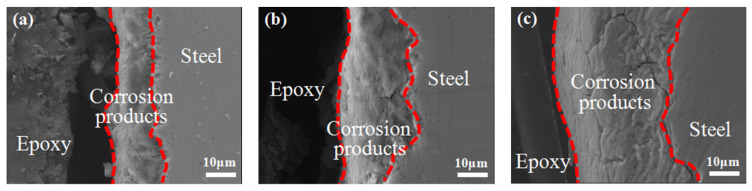
Cross-sectional morphology of corrosion products after different time corrosion: (**a**) 72 h; (**b**) 288 h; (**c**) 600 h.

Figure 5 shows the cross-sectional morphology of the corrosion products after different corrosion times. The thickness of corrosion product film increases obviously with the prolongation of corrosion time. After 72 h of corrosion, only a thin layer of corrosion products is produced on the substrate surface, which cannot effectively prevent the diffusion of corrosive ions in the medium to the substrate. It can be seen from the section morphology after 288 h corrosion that the substrate surface under the rust layer of medium-Mn steel is uneven and seriously concave, which indicates that the pitting corrosion of the steel matrix is serious in Cl^-^-rich simulated seawater corrosion medium. This uneven surface is actually due to the selective corrosion of the austenite phase, which is enriched with Mn, as the local higher dissolution rate of Mn in austenite is responsible for this behavior [28]. After 600 h of corrosion, the surfaces of sample lost seriously, and a very thick rust layer was also formed on the substrate surface, but there are a few slender cracks in the dense and thick rust layer. Studies have shown that the selective permeability of cations in the rust layer makes the anions blocked at the anion–cation interface; the anions concentrate at the interface and start to diffuse from the interface to the outer rust layer at higher concentrations, leading to the formation of cracks at the anion–cation interface [29]. In addition, with the evolution of corrosion products, the volume change caused by oxide formation is also the cause of crack formation.

### 3.5. Element Distribution and Valence State

**Figure 6:**
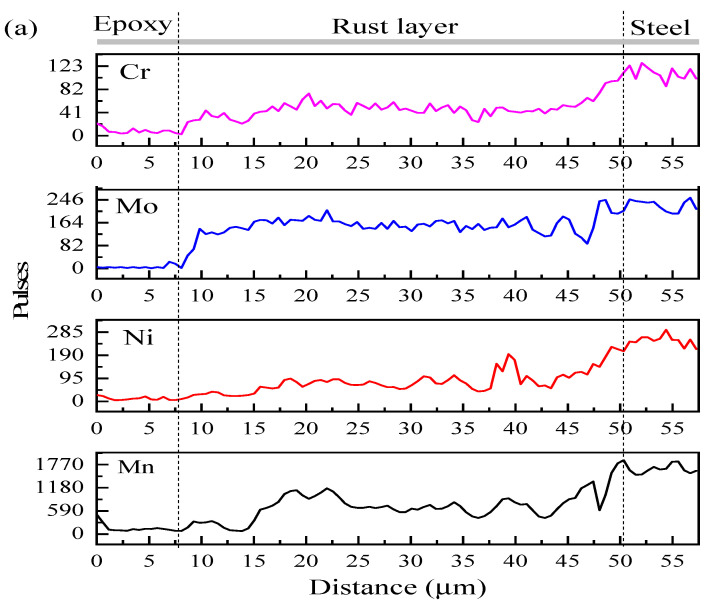

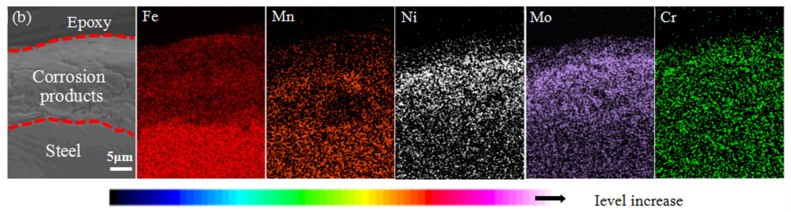
The distribution of elements in corrosion products of test steel: (**a**) the results of EDS line scan for 24 h; (**b**) results of EDS surface scan for 600 h.

Figure 6 shows the elemental distribution of corrosion products after 24 h and 600 h corrosion. The results show that the alloying elements Mn, Ni, Cr, and Mo are enriched or depleted to varying degrees, the enriched and depleted areas change significantly with the extension of corrosion time. In the corrosion product film of tested steel at the initial corrosion stage, the Mn content is much higher than Ni, Cr, and Mo, and the enrichment in the outer rust layer is significant. Mo is evenly distributed in the rust layer, and Ni is higher in the inner rust layer. Since the activity of Mn is higher than that of Fe, Ni, and other alloy elements, the manganese-rich phase is easy to lose electrons as the anode in the corrosion electrochemical system, which promotes the dissolution of anode metal elements [30] and then intensifies the corrosion rate of test steels in the initial corrosion stage. In the corrosion products of test steel at the late corrosion stage, Ni is obviously enriched in the middle of the corroded layer, Mn appears in a depleted area on both the inside and outside of the corroded layer, Cr and Mo is evenly enriched in the whole cross-sectional area of the rust layer. Mo oxides can form a stable passive film, reduce the impact of chloride ion on corrosion, and play a role in corrosion inhibition [31,32]. Ni enrichment can change the properties of corrosion products and improve their corrosion potential [33,34]. On the other hand, it helps to form spinel oxides and improve the denseness of the corroded layer, which in turn enhances the protection ability of corrosion products. 

**Figure 7:**
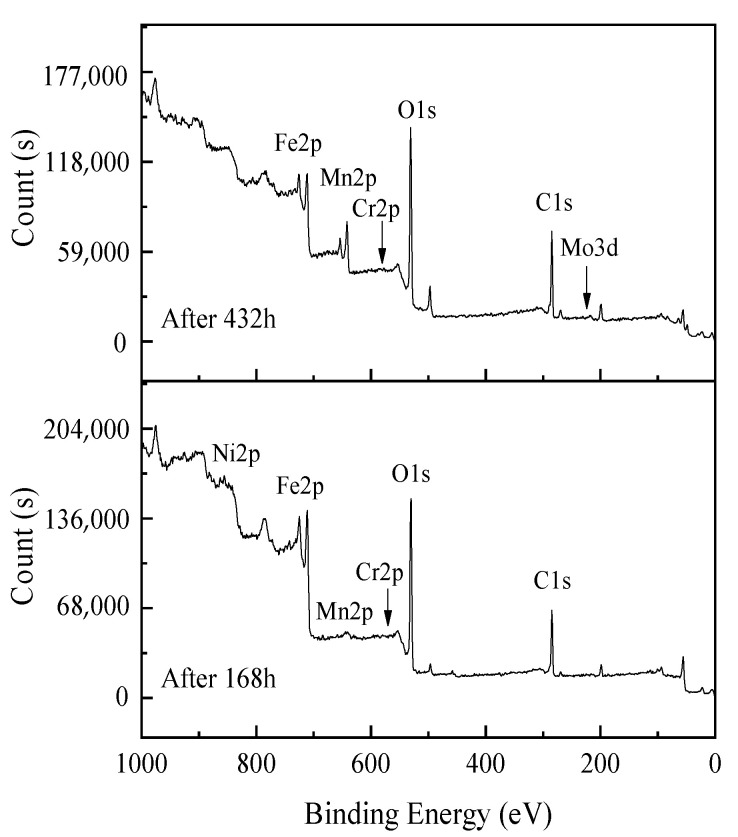
XPS survey spectra of corrosion products formed after 168 h and 432 h corrosion.

**Figure 8:**
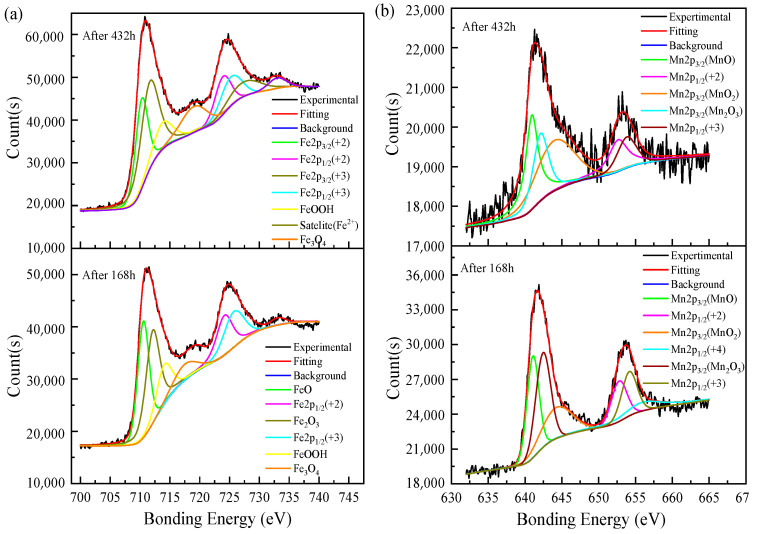
XPS patterns of corrosion products of tested steel after corrosion testing times of 168 h and 432 h: (**a**) Fe2p; (**b**) Mn2p.

The XPS survey spectra of the corrosion products formed after 168 h and 432 h corrosion tests are shown in Figure 7. The peaks of Fe2p, Mn2p, Ni2P, Mo3d, Cr2p, O1s, and C1s were detected in the spectra of corrosion products, while the other elements were not easily detected due to their low content. The O1s peak shows that the sample oxidation is serious; combined with the corrosion product XRD results, O and Fe, the Mn elements formed by oxides on the corrosion evolution process are significant. As shown in Figure 8, the XPS spectra of Fe2p and Mn2p in the corrosion products were fitted with avantage software to determine the main components. After 168 h of corrosion, the peaks at 710.60 eV and 724.15 eV in the Fe2p spectrum correspond to Fe2p_3/2_ and Fe2p_1/2_ of Fe (II), the peaks at 712.20 eV and 726.08 eV belong to Fe2p_3/2_ and Fe2p_1/2_ of Fe (III), and the peaks at 714.78 eV and 718.18 eV confirmed the corrosion and presence of Fe (+3) oxides and hydroxyl oxides (FeOOH) in the products. After 432 h of corrosion, the deconvoluted peaks at 711.89 eV, 713.67 eV, and 719.31 eV in the Fe2p_3/2_ spectrum confirmed that Fe_2_O_3_, FeOOH, and Fe_3_O_4_ were mainly present in the corrosion products, and the peak of Fe2p_1/2_ was at 728.35 eV. With the corrosion durations, the peak of FeO in the corrosion product decreased, while the FeOOH and Fe_3_O_4_ peaks are enhanced. The peaks of Mn2p_3/2_ at 640.93 eV and 642.51 eV in the spectrum of Mn2p measured after a 168 h corrosion test of the sample steel confirmed the presence of MnO and Mn_2_O_3_ in the corrosion products, and the peaks at 652.91 eV and 654.10 eV corresponded to Mn2p_1/2_ of Mn (+2) and Mn (+3). After 432 h of corrosion, the peaks of the fitting spectra of Mn2p at 640.93 eV, 642.24 eV, and 644.62 eV are attributed to Mn2p_3/2_ of the three valence states of Mn, indicating the simultaneous presence of three manganese oxides of MnO, Mn_2_O_3_, and MnO_2_ in the terminal corrosion products.

**Figure 9:**
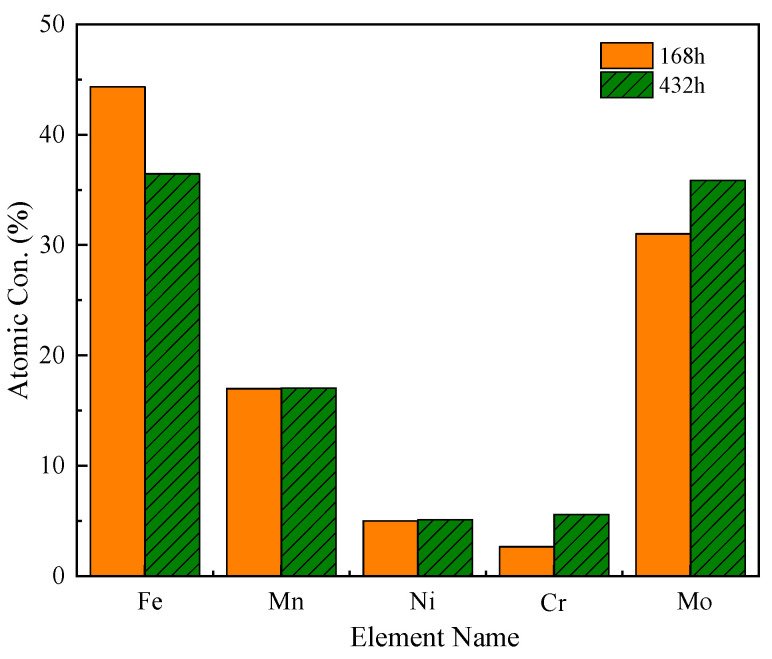
Atomic concentration of main elements in corrosion products after 168 h and 432 h corrosion.

The atomic concentrations of the elements in the corrosion products after 168 h and 432 h corrosion are shown in Figure 9. Using XPS for semi-quantitative analysis of the main elements in corrosion products, the atomic concentration (*C_A_*) of any element in the corrosion product can be calculated by Equation (2):

(2) $$C_{A}={\left(At\;\%\right)}_{A}=\frac{N_{A}}{\sum_{i}^{A}N_{i}}\times 100\%=\frac{I_{A}/S_{A}}{\sum_{i}^{A}I_{i}/S_{i}}\times 100\%$$

where $N_{i}$ represents the normalized area, $I_{i}$ represents the area of the corresponding spectral peak of the element, and $S_{i}$ represents the relative sensitivity factor of the element. 

The results show that the atomic concentrations of the alloying elements Mn, Ni, Cr, Mo, and Fe in the corrosion products change significantly with the corrosion durations. In the corrosion products of the sample at the two corrosion stages, the concentration of Fe decreased significantly, the concentration of Mn and Ni did not change significantly, and the concentration of Cr and Mo elements increased significantly after 432 h of corrosion, indicating that the corrosion resistance of the late corrosion products of medium-Mn steel increased mainly with the enrichment of Cr and Mo, which have a close relationship. 

### 3.6. Electrochemical Behavior

**Figure 10:**
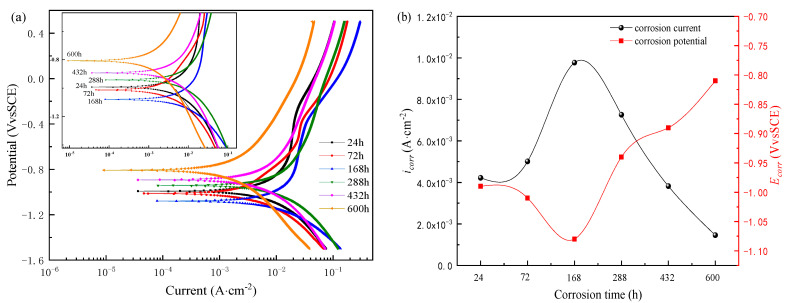
Electrochemical polarizations of test steels after corrosion for different times: (**a**) polarization curves; (**b**) variation of corrosion current density and corrosion potential with time.

Figure 10 shows the potentiodynamic polarization curve of the corrosion product film formed on the surface of the sample steel after different corrosion times. The results show that the cathodic polarization curves do not exhibit an oxygen diffusion zone but show typical characteristics of hydrogen release control. The anodic polarization curves of the corrosion product film that formed in the early corrosion stage show some regions in which the change of corrosion current density suddenly slows down with the positive shift of potential. It means that the corrosion product film formed on the surface of the substrate in this zone plays a temporary protective role. At the initial corrosion stage, the corrosion products are very thin, with uneven adhesion and incomplete film formation. With the positive shift of scanning potential, the corrosion product film is easy to be broken down, the substrate surface is activated and dissolved again, and the corrosion current continues to increase. In the late corrosion stage, the corrosion product film is thicker and more uniform, so the anodic polarization curve also did not appear to have any corrosion current density with the potential change of the obvious fluctuation zone. Electrochemical parameters corresponding to polarization curve, as shown in Table 2. Tafel fitting results show that with the dry–wet cyclic corrosion, the testing time is prolonged. The corrosion current density (***i***_corr_) of the corrosion product film first increases and then decreases, and the corrosion potential (***E***_corr_) first moves negative and then gradually shifts to positive. It shows that corrosion resistance and thermodynamic stability of the corrosion product film both increase with the corrosion time. This is also consistent with the results of the previous kinetic calculations. 

**Figure 11:**
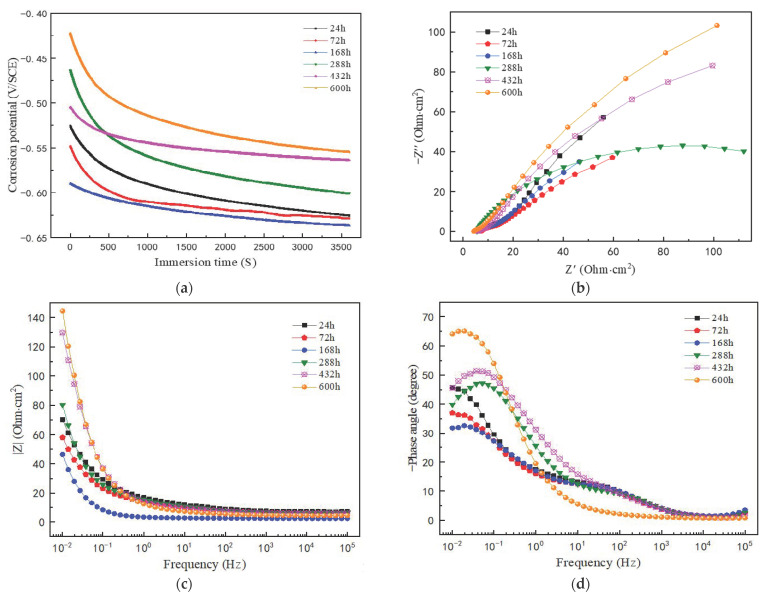
Nyquist and Bode plots of medium-Mn steel in simulated seawater medium after corrosion at different times: (**a**) OCP for 1 h; (**b**) Nyquist plot; (**c**) impedance mode vs. frequency plot; (**d**) phase angle vs. frequency plot.

Figure 11 shows the Nyquist and Bode plots of the electrochemical impedance spectra of the corrosion product film formed on the surface of the sample steel after different corrosion times. Firstly, a 1 h open circuit potential test shall be conducted on the samples with corrosion product film after dry–wet cycle corrosion for different times, as shown in Figure 11a. The results show that the potential curves of the corrosion product film that formed at each corrosion stage tend to be stable after stabilizing for one hour, and the corrosion potential value first moves negative and then positive with the extension of corrosion testing time. It shows that the thermodynamic stability of the corrosion product film increases with the extension of dry–wet corrosion time; that is, the corrosion resistance trend of the corrosion product film increases gradually. From Figure 11b, it can be seen that the Nyquist impedance spectra of the corrosion product films formed in each corrosion stage show similar characteristics, which are capacitive arcs in the first quadrant. The size of the capacitive arc radius is generally considered to represent the charge transfer resistance of the corrosion process, and a larger radius indicates a higher charge transfer resistance [35,36,37]. The corrosion product film of the capacitive arc radius of resistance with the corrosion time extends the first decrease and then gradually increases, indicating that the reaction resistance of the corrosion process gradually increased and the corrosion rate decreased. From Figure 11c, it can also be seen that with the corrosion time extended, the impedance modulus of corrosion product film increases gradually at low frequency. As reflected in the relationship between the frequency and phase angle of the Bode diagram in Figure 11d, each curve presents a peak at low frequency and a slightly raised valley at high frequency. So, it can be determined that there are two time constants for EIS of a steel rust layer in each corrosion stage.

**Figure 12:**
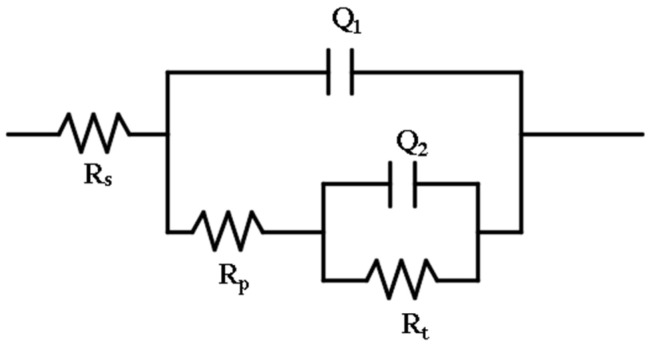
Test steel electrode surface corrosion process EIS equivalent simulation circuit diagram.

The impedance capability of the corrosion product film formed after corrosion for different times was evaluated; the equivalent circuit (EEC) shown in Figure 12 was used to do the impedance fitting analysis, with the equivalent circuit code as *R_s_*(*Q*_1_(*R_p_*(*Q*_2_*R_t_*))).

Table 3 shows the fitting parameter values of the electrochemical impedance spectrum of the corrosion product film after corrosion for different times. *R_s_* is corrosion electrolyte resistance, *Q*_2_ is double-layer capacitance at the interface between the electrolyte and corrosion product film, *R_t_* is the resistance to charge transfer, i.e., the amount of resistance to ion migration under the action of an electric field, *Q*_1_ is the non-ideal capacitance of the corrosion product on the surface of the specimen, *R_p_* is the resistance of the corrosion product on the surface of the specimen steel: the larger its value, the more stable the generated corrosion product film. The results show that the charge transfer resistance *R_t_* and corrosion product resistance *R_p_* increase as the corrosion testing time is prolonged. It shows that the protective effect of the corrosion product film is gradually enhanced in the dry–wet alternating corrosion environment.

## 4. Conclusions

The corrosion rate, corrosion product composition, surface and cross-section morphology, element distribution and valence state, polarization curve, and electrochemical impedance (EIS) of test medium-Mn steel after dry–wet cyclic corrosion for different times were detected and analyzed, and the following conclusions were reached:

1. The corrosion rates increase first; then, they decrease and tend to be stable. At the initial stage of corrosion, the cathodic process includes oxygen reduction and the reduction of rust layer components; the strong or weak change of these two effects is the main reason for the increase in initial corrosion rate, and the turning of velocity is caused by the compactness or physicochemical properties of the rust layer. With the extension of corrosion time, the rust layer changes from loose to dense.
2. In the dry–wet cyclic corrosion environment, the corrosion products of the sample mainly include Fe_x_O_y_, Mn_x_O_y_, γ-FeOOH, and a small amount of (Fe,Mn)_x_O_y_, and the valence state of iron compounds and manganese compounds in different corrosion stages changed obviously.
3. The corrosivity of the rust layer is related to the enrichment of alloy elements. In the initial corrosion products, the content of Mn is much higher than that of alloy elements Ni, Mo, and Cr, and it is significantly enriched in the outer rust layer, which promotes the process of corrosion electrochemical reaction. The content of Ni in the inner rust layer is high. The element semi-quantitative analysis of corrosion products shows that the atomic concentrations of Cr and Mo increase significantly in later corrosion products, indicating that the dense isolation layer formed by alloy element compounds in corrosion products is the main factor to improve the protection ability of corrosion product film at the end corrosion stage of the sample.
4. With corrosion durations, the corrosion current density (*i*_corr_) of the corrosion product film first increased and then decreased, and the corrosion potential (*E*_corr_) first becomes negative and then gradually shifts to positive. The impedance fit also showed an increase in both corrosion product resistance (*R_p_*) and charge transfer resistance (*R_t_*), indicating a gradual increase in corrosion product film stability.

## Figures and Tables

**Table 1:** Annual corrosion rates of test steels.

Corrosion Durations (h)		24	72	168	288	432	600
corrosion rate (mm/y)	9Ni steel medium Mn steel high Mn steel	0.7169 0.7105 0.8266	0.9600 0.9691 1.3813	0.6696 1.4230 1.7250	0.6543 1.0818 0.8747	0.6340 0.9639 0.7020	0.6025 0.9274 0.6240

**Table 2:** Electrochemical parameters corresponding to polarization curve.

Corrosion Durations	24 h	72 h	168 h	288 h	432 h	600 h
***E***_corrsion_ (V) ***i***_corrsion_ (A/cm^2^) ***b***_a_, V/dec ***b***_c_, V/dec	−0.99 4.22 × 10^−3^ 0.077 −0.057	−1.01 5.01 × 10^−3^ 0.069 −0.049	−1.08 9.77 × 10^−3^ 0.042 −0.02	−0.94 7.26 × 10^−3^ 0.05 −0.046	−0.89 3.82 × 10^−3^ 0.063 −0.049	−0.81 1.46 × 10^−3^ 0.041 −0.027

**Table 3:** Fitting parameters of EIS shown in Figure 11 by the equivalent circuit in Figure 12.

Parameters	*R_s_* (Ω·cm^2^)	*Q*_1_ (F·cm^–2^)	*N*	*R_p_* (Ω·cm^2^)	*Q*_2_ (F·cm^–2^)	*n*	*R_t_* (Ω·cm^2^)
72 h 288 h 600 h	6.955 5.065 4.364	9.248 × 10^−3^ 2.650 × 10^−3^ 1.315 × 10^−2^	0.862 0.733 0.707	8.857 9.182 17.26	5.748 × 10^−2^ 2.604 × 10^−3^ 2.679 × 10^−2^	0.705 0.719 0.697	252.7 353.6 654.5

## Data Availability

Data available in a publicly accessible repository.
